# *Fusobacterium* Is Associated with Colorectal Adenomas

**DOI:** 10.1371/journal.pone.0053653

**Published:** 2013-01-15

**Authors:** Amber N. McCoy, Félix Araújo-Pérez, Andrea Azcárate-Peril, Jen Jen Yeh, Robert S. Sandler, Temitope O. Keku

**Affiliations:** 1 Center for Gastrointestinal Biology and Disease, University of North Carolina at Chapel Hill, Chapel Hill, North Carolina, United States of America; 2 Division of Gastroenterology and Hepatology, Department of Medicine, University of North Carolina at Chapel Hill, Chapel Hill, North Carolina, United States of America; 3 Microbiome Core Facility, Center for Gastrointestinal Biology and Disease and Department of Cell and Molecular Physiology, University of North Carolina at Chapel Hill, Chapel Hill, North Carolina, United States of America; 4 Departments of Surgery and Pharmacology, Lineberger Comprehensive Cancer Center, University of North Carolina at Chapel Hill, Chapel Hill, North Carolina, United States of America; Baylor University Medical Center, United States of America

## Abstract

The human gut microbiota is increasingly recognized as a player in colorectal cancer (CRC). While particular imbalances in the gut microbiota have been linked to colorectal adenomas and cancer, no specific bacterium has been identified as a risk factor. Recent studies have reported a high abundance of *Fusobacterium* in CRC subjects compared to normal subjects, but this observation has not been reported for adenomas, CRC precursors. We assessed the abundance of *Fusobacterium* specie*s* in the normal rectal mucosa of subjects with (n = 48) and without adenomas (n = 67). We also confirmed previous reports on *Fusobacterium* and CRC in 10 CRC tumor tissues and 9 matching normal tissues by pyrosequencing. We extracted DNA from rectal mucosal biopsies and measured bacterial levels by quantitative PCR of the 16S ribosomal RNA gene. Local cytokine gene expression was also determined in mucosal biopsies from adenoma cases and controls by quantitative PCR. The mean log abundance of *Fusobacterium* or cytokine gene expression between cases and controls was compared by t-test. Logistic regression was used to compare tertiles of *Fusobacterium* abundance. Adenoma subjects had a significantly higher abundance of *Fusobacterium* species compared to controls (p = 0.01). Compared to the lowest tertile, subjects with high abundance of *Fusobacterium* were significantly more likely to have adenomas (OR 3.66, 95% CI 1.37–9.74, p-trend 0.005). Cases but not controls had a significant positive correlation between local cytokine gene expression and *Fusobacterium* abundance. Among cases, the correlation for local TNF-α and *Fusobacterium* was r = 0.33, p = 0.06 while it was 0.44, p = 0.01 for *Fusobacterium* and IL-10. These results support a link between the abundance of *Fusobacterium* in colonic mucosa and adenomas and suggest a possible role for mucosal inflammation in this process.

## Introduction

The human intestinal microbiota inhabits a complex and diverse environment populated by hundreds of different bacterial species. The number of bacterial cells in the gut exceeds all other eukaryotic cells in the human body by a factor of 10 [1], [2]. These bacteria are regulated in the gut by the mucosal immune system, which is made up of a complex network of functions and immune responses aimed at maintaining a cooperative system between the intestinal microbiota and the host [1]. In a healthy gut these bacteria maintain homeostasis with the host. However, when an imbalance, or bacterial dysbiosis, occurs in the gut, the host may experience inflammation and a loss of barrier function [3], [4]. Bacterial dysbioses have been linked to several diseases including ulcerative colitis, Crohn’s disease [5]–[7] and colorectal cancer (CRC) [8], [9]. Current research is focused on identifying key players in this imbalance as well as their specific contribution to colorectal carcinogenesis.

No single bacterial species has been identified as a risk factor for CRC, but recent studies report an increase in the abundance of *Fusobacterium* in human colorectal tumors compared to controls [8], [10], [11]. These studies suggest that *Fusobacterium* may be associated with the later stages of CRC, but it is unknown if they play a role in the early stages of colorectal carcinogenesis. While the causes of colorectal cancer are not fully known, it is becoming increasingly clear that the gut microbiota provide an important contribution [12].

We evaluated whether *Fusobacterium nucleatum* in normal rectal mucosal biopsies was associated with colorectal adenomas. We also examined a potential association between local inflammation and abundance of *Fusobacterium* in adenoma cases and non-adenoma controls. We found that *Fusobacterium* was more abundant in adenoma cases than controls. We observed significant positive correlation between IL-10 and TNF-α gene expression and abundance of *Fusobacterium* species in cases. Validation experiments were performed using CRC tissue and matching normal tissue to confirm previous reports of an association between CRC and *Fusobacterium*.

## Results

### Studies in Normal Rectal Mucosa of Adenoma and Non-adenoma Subjects

#### *Fusobacterium* abundance is higher in adenoma cases compared to controls

**Figure 1:**
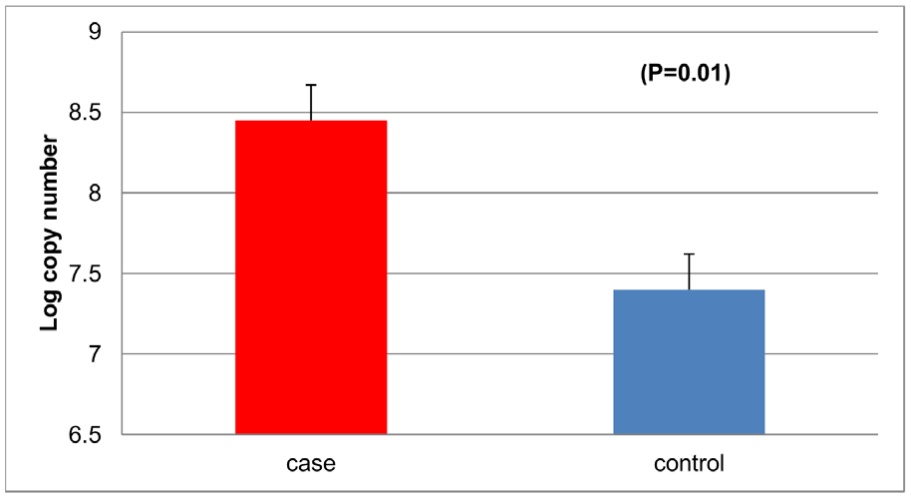
Abundance of *Fusobacterium* in rectal mucosal biopsies from adenoma cases and non-adenoma controls. qPCR results show that *Fusobacterium* is more abundant in cases than controls.

We evaluated *Fusobacterium* species in normal mucosal biopsies from 115 subjects, 48 cases and 67 controls by qPCR. Subject characteristics are shown in Table 1. All subjects were similar in age with cases having a mean age of 56.38±0.92, and controls 55.90±0.88 years. There were no significant differences between adenoma cases and non-adenoma controls for several risk factors evaluated including alcohol intake, caloric intake, waist-hip ratio, body mass index and total fat intake. Abundance of *Fusobacterium* species was significantly higher in adenoma cases compared to controls (mean log copy number and standard error, cases, 8.44±0.38; controls 7.40±0.22 p = 0.01) (Fig. 1). Compared to those with low abundance of *Fusobacterium,* those with high abundance of *Fusobacterium* were more likely to be adenoma cases (p-trend = 0.005) (Table 2). We also assessed the correlation between *Fusobacterium* abundance and the frequency and size (small, medium, large) of adenomas among cases. There was no significant correlation between *Fusobacterium* species and adenoma size (Table S1) or number of adenomas (r = −0.08, p = 0.57).

**Table 1:** Characteristics of Study Participants.

Characteristic	Case (n = 48)	Control (n = 67)	P-value
Age (years, mean, se)	56.38±0.92	55.90±0.88	0.71
Waist-Hip ratio(mean, se)	0.94±0.01	0.91±0.01	0.14
Body Mass Index(kg/m^2^, mean, se)	27.40±0.61	27.04±0.66	0.70
Alcohol Intake(g/day, mean, se)	12.65±1.94	21.17±8.88	0.41
Calories(kcal/day, mean, se)	2108.70±114.78	2140.38±144.0	0.87
Total Fat intake(g/day, mean, se)	82.36±5.31	79.36±4.78	0.67
Red meat intake(oz/day, mean, se)	1.59±0.17	1.36±0.14	0.30
Dietary Fiber(g/day, mean, se)	23.03±1.28	25.58±1.76	0.27

**Table 2:** Association between *Fusobacterium* abundance and colorectal adenomas.

Categories*	Case (n = 48)	Control (n = 67)	OR (95% CI)**
Tertile 1	8	23	Reference
Tertile 2	12	22	1.57 (0.54–4.57)
Tertile 3	28	22	3.66 (1.37–9.74)
P trend			

* The abundance of *Fusobacterium* among control subjects were used to generate tertile cut-off. The lowest tertile of *Fusobacterium* abundance was considered as the reference.

** Odds ratio and 95% confidence interval.

Compared to subjects with a low copy number, subjects with high abundance of *Fusobacterium* are more likely to be adenoma cases than controls.

#### Localization of *Fusobacterium* in colonic mucosal by FISH analysis

**Figure 2:**
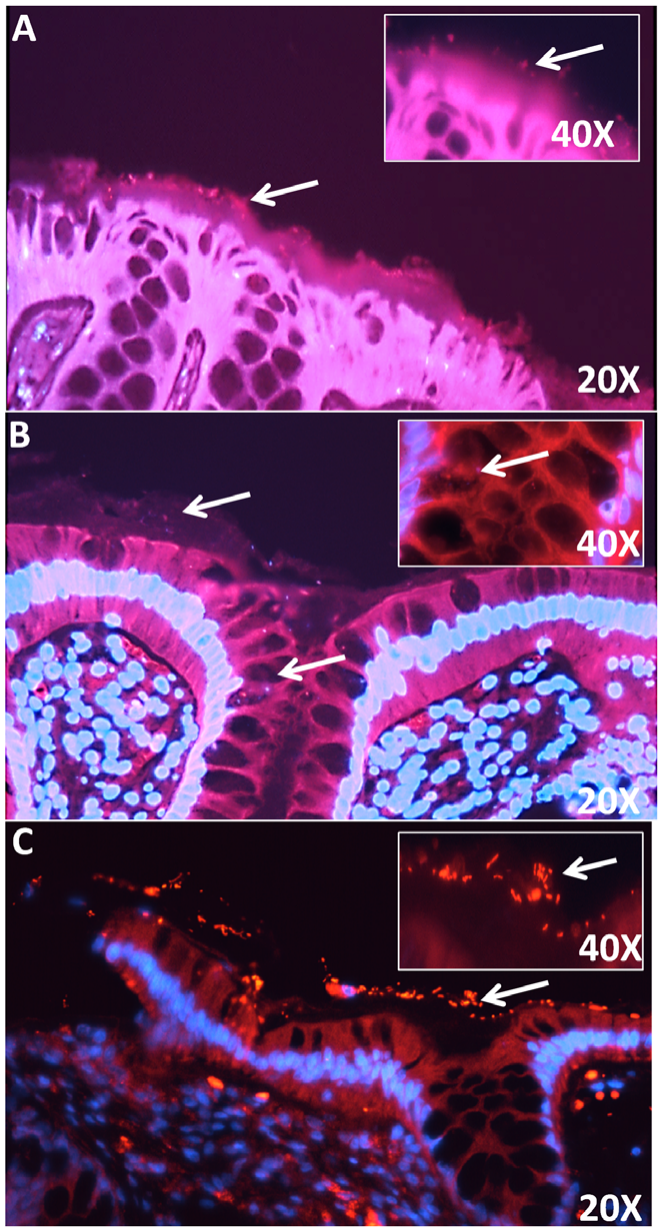
Representative fluorescence *in situ* hybridization targeting *Fusobacterium sp.* in colorectal mucosal biopsy sections using bacterial 16S rRNA probes. Fig. 2A–B are composite images of Cy3 and DAPI views of sections hybridized with a *Fusobacterium*-specific probe. *Fusobacterium* species is localized within the mucus layer of colorectal sections (A) 20X and 40X. *Fusobacterium* species is localized within the crypts of colorectal section (B) 20X and 40X. Fig. 2C (20X and 40X) is a positive control and shows sections stained with general bacteria probe (Eub 388). General bacteria, including most *Eubacteria species,* are localized to the mucus layer above the epithelium. White arrows point to bacteria either in mucus layer above the colonic epithelium or within the crypt.

We Observed that *Fusobacterium* was over-represented in adenoma cases compared to non-adenoma controls, therefore, we performed histological evaluation by Fluorescence *in situ* Hybridization (FISH) using a *Fusobacterium*-specific probe to localize *Fusobacterium* in colonic mucosal tissue sections (Fig. 2a and 2b). The results show that *Fusobacterium* was localized in the mucus layer above the epithelium as well as within the colonic crypts. A general bacterial probe was also used as a positive control (Fig. 2c). Results confirm the presence of bacteria in the mucus layer.

#### There is a significant positive correlation between *Fusobacterium* species abundance and local inflammation in adenoma cases

**Figure 3:**
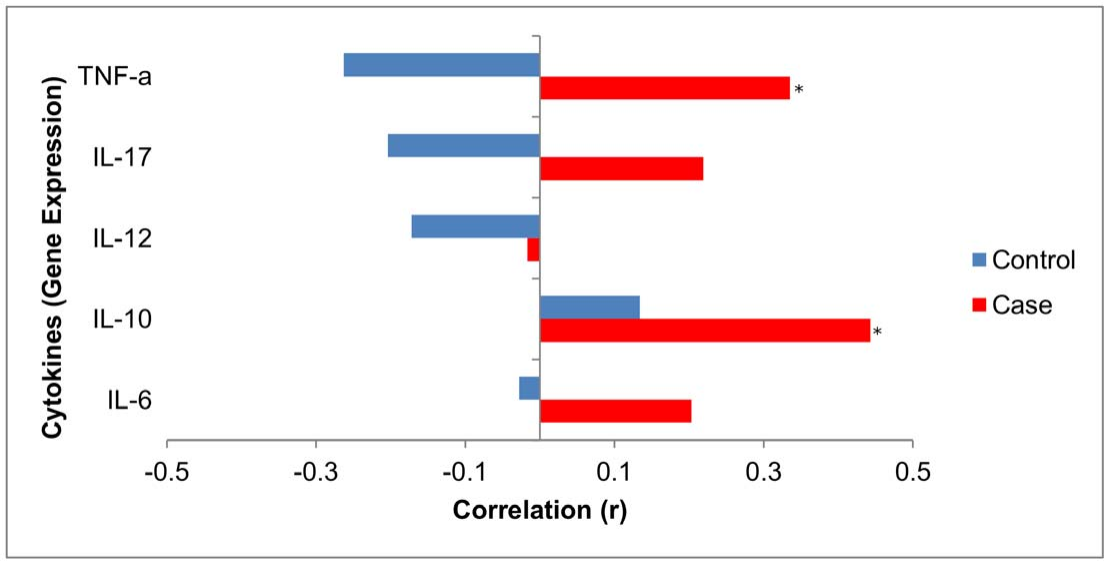
Correlations between *Fusobacterium* abundance and local cytokine gene expression in adenoma cases and non-adenoma controls. Results suggest a significant positive correlation between *Fusobacterium* abundance and local inflammation in cases but not controls. The Correlations were significant for IL-10 (r = 0.44, p = 0.01) and TNF-α (r  = 0.33, p = 0.06). *p<0.05.

Correlation of local inflammatory cytokine gene expression and *Fusobacterium* species abundance was analyzed separately for adenoma cases and non-adenoma controls. Analysis of cytokines IL-6, IL-10, IL-12, IL-17 and TNF-α and *Fusobacterium* was observed to have a significant positive correlation with local inflammation in cases, but not controls (Fig. 3). A significant positive correlation was found between abundance of *Fusobacterium* species and IL-10 (r  = 0.443 p  = 0.01). The correlation for TNF-α (r = 0.335 p = 0.06) was borderline significant. Although the correlations for IL-6 and IL-17 were positive, they did not reach statistical significance.

### Confirmatory Studies in Colorectal Cancer

#### Pyrosequencing analysis of 16s rRNA gene in colorectal cancer (CRC) tissue and matched normal colonic tissue revealed higher *Fusobacterium* species abundance in CRC compared to normal tissue

**Figure 4:**
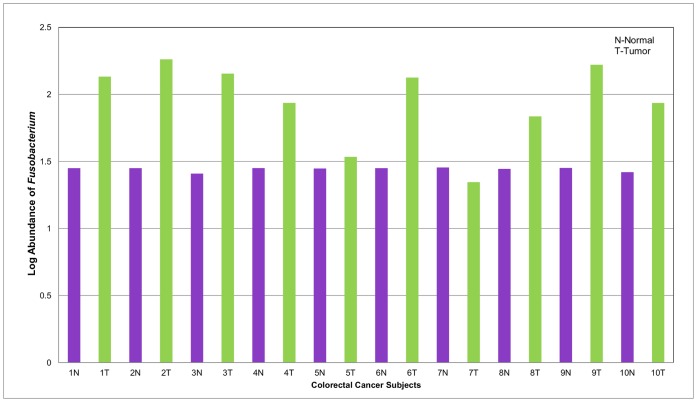
Log Abundance of *Fusobacterium* in matched normal colon and colorectal cancer tissue. *Fusobacterium* abundance was evaluated in DNA samples from normal colon and tumor tissue by qPCR using *Fusobacterium-*specific primers. Results suggest that *Fusobacterium* is increased in colon cancer tissue compared to normal tissue (t-test p = 0.0005).

Previous studies reported an association between *Fusobacterium* species and colorectal cancer [8], [10], [11]. We reproduced these results by conducting high-throughput pyrosequencing analysis on 19 matched samples, 10 CRC tissues and 9 non-malignant matched controls from adjacent mucosa. All subjects were Caucasian and predominantly female, with ages ranging from 37–78 years. High-throughput sequencing revealed differences in abundance and richness in CRC compared to normal tissue. We identified 13 phyla, 24 classes and 176 bacteria genera. Overall, Shannon diversity and richness were higher in the CRC samples than matched normal tissue. Abundance of individual bacteria varied between groups. We observed reduced abundance of Bacteroidetes in CRC tissue compared to normal colon tissue, however, the distribution of the phylum Fusobacteria was higher in CRC tissue. The results showed a higher abundance of *Fusobacterium* in the CRC tissue compared to normal tissue. (Fig. 4).

#### qPCR validation of *Fusobacterium* species in colorectal cancer patients

qPCR analysis of *Fusobacterium* species in 10 CRC and 9 matching normal control tissues revealed a significant increase in abundance among colorectal cancer tissue compared to normal tissue, confirming previously reported results of higher *Fusobacterium* abundance in CRC patients. We also evaluated the relationship between CRC characteristics such as tumor location, treatment and *Fusobacterium* abundance. We did not observe significant associations for most of the tumor characteristics; however, we observed higher abundance of *Fusobacterium* species in the sigmoid than right side tumor location (Table 3). We further validated the pyrosequencing results by qPCR and observed significantly positive correlation between the two methods (r = 0.76, p = 0.0001).

**Table 3:** Relationship between *Fusobacterium* and colorectal tumor characteristics.

Variable	*Fusobacterium*(copy #, mean, se)	P-value
Tumor Location		
Right	1.82±0.13	
Transverse	1.94±0.09	NS
Sigmoid	2.21±0.31	0.04 Sigmoid vs. Right
Stage		
T-2	1.83±0.29	
T-3	1.98±0.11	0.56
Adjuvant Therapy		
No	2.16±0.03	0.20
Yes	2.01±0.10	

## Discussion

The human gut microbiota has been shown to have a dynamic and observable impact on the human host [4], [13]. While many of these bacteria are commensal and facilitate the maintenance of a healthy and functioning gastrointestinal tract, current research has shown that interactions between the host and the bacteria colonizing the gut can contribute to various diseases including colorectal carcinogenesis [12]–[15]. In particular, bacterial dysbiosis in the gut has been implicated in colorectal neoplasia, although no specific bacteria or bacterial signatures have been identified for colorectal adenomas [8], [9]. We evaluated the abundance of *Fusobacterium* in relation to colorectal adenomas in a case-control study and found that compared to controls, cases had significantly higher abundance of *Fusobacterium.*

There has been a recent focus on *Fusobacterium,* Gram-negative bacteria that usually colonize the oral cavity [10], [16]. Several groups have identified *Fusobacterium* in tumors of patients with colorectal carcinoma [8], [10], [17]–[19] and reported that the tumor tissue was characterized by a higher abundance of *Fusobacterium* than that of the normal colon. These results suggest *Fusobacterium* as a potential biomarker for colorectal carcinogenesis. However, it is not known whether *Fusobacterium* is associated with adenomas, early precursors of CRC. We observed significant differences in bacterial abundance between adenoma versus non-adenoma subjects and found that there was a strong positive correlation between high abundance of *Fusobacterium* and the presence of colorectal adenomas (p = 0.01). In particular those with high levels of *Fusobacterium* had about three and half fold increased risk of adenomas. As a CRC precursor, adenomas have become increasingly important in the study of colorectal carcinogenesis. Our results suggest that the changes in gut microbiota are associated with the earliest stages of tumor development [20]. Our results for colorectal adenomas and increased *Fusobacterium* levels are similar to previously reported studies involving *Fusobacterium* and colorectal cancer [8], [10], [11]. We validated the previously reported association between *Fusobacterium* and colorectal carcinoma in a set of matched CRC tumor and normal human colon tissue samples. Using both pyrosequencing and qPCR analysis of the 16S bacterial rRNA gene we were able to successfully reproduce these published results. We found that among CRC tumors and matched controls, *Fusobacterium* abundance was significantly higher in tumor tissue based on both qPCR as well as pyrosequencing analysis, with a significant correlation between both methods (r = 0.76, p = 0.0001).

We and others observed a difference in *Fusobacterium* abundance between the colorectal tumor and adjacent non-neoplastic tissue [10], [11], however, it would also be beneficial in future studies to assess the actual adenomas specifically, compared to normal mucosa. Our findings raise several important questions. With regard to colorectal adenomas and cancer, is *Fusobacterium* causative agent or an opportunistic colonizer? Does *Fusobacterium* act alone or in concert with other bacteria, viruses or fungi to promote CRC? Are there specific changes in the colonic environment that contribute to increased abundance of *Fusobacterium* in carcinogenesis? What are the mechanisms involved in this process? These questions will need to be addressed in future studies, particularly in animal models of adenomas and CRC to uncover whether *Fusobacterium* is a causative agent or opportunistic colonizer of colorectal tissue. Several potential factors such as host inflammation and altered gut environment (pH, bile acids, presence of adenoma or cancer) may contribute to the relationship between *Fusobacterium* and adenomas. These are addressed below.

Inflammation is a known risk factor for CRC [21], [22]. Interestingly, intestinal inflammation has been repeatedly linked to the gut microbiota [23], [24]. Commensal gut bacteria interact with the host in a symbiotic way to facilitate the operation of the intestinal immune system. However, as reported by several studies, bacterial dysbiosis may contribute to dysregulation of the immune system and mucus production in the gut, ultimately disrupting the delicate homeostatic relationship between commensal bacteria and the human host [3], [25], [26]. Uronis *et al.* [26] successfully demonstrated a link between the microbiota, intestinal inflammation and increased risk of colitis-associated colorectal cancer (CAC) in a mouse model. More specifically, *Fusobacterium* has been implicated as a pro-inflammatory pathogen [25], [27], [28] and has been found in a higher abundance of IBD patients [18].

*Fusobacterium sp.* have been found to flourish primarily in the oral cavity where they have been observed to behighly invasive [18], [29] and adherent [30]–[32]. The ability of *Fusobacterium* to attach to mucosal surfaces [28] makes it an ideal candidate to study in relation to host immunity and adenomas. Given the reports of previous studies that link *Fusobacterium* with inflammatory bowel diseases (IBD), we evaluated its relationship with several inflammatory markers. Specifically, we assessed mRNA expression of mucosal inflammatory cytokines IL-6, IL-10, IL-12, IL-17 and TNF-α in normal rectal biopsies and correlated their expression levels with abundance of *Fusobacterium* species in adenoma and non-adenoma subjects. We observed a positive correlation between the gene expression of several local cytokines and *Fusobacterium* species in adenoma cases, but not in controls. Similar to previously published findings [25], we saw a significant association between increased abundance of *Fusobacterium* and TNF-α. Dharmani *et al.* observed that the presence of invasive *F. nucleatum* strains correlated with increased TNF-α expression in IBD patients as well as experimental models [25]. Therefore, taking into consideration the observations that *Fusobacterium* is invasive and adherent, its link with IBD, its increased abundance in adenoma cases compared to controls, as well as a positive correlation with local inflammation, one could suggest that *Fusobacterium* may contribute to increased mucosal inflammation in adenoma subjects. However, we also detected a significant positive correlation between *Fusobacterium* and IL-10 expression in adenoma cases. While this observation with IL-10 is interesting, it highlights the complex and multi-factorial relationship between the host and its enteric intestinal bacteria.

The human host and the gut bacteria share a complex symbiotic relationship in which they both exert considerable influence on each other. Tumors are known to acidify their microenvironment and cause fluctuations in pH, thus the presence of an adenoma or cancer could lead to stress of the gut environment [33]. The presence of adenoma or cancer could change the luminal environment by altering levels of bile acids, pH, nutrient levels and redox potential, all of which could impact the gut microbial community and favor overgrowth of opportunistic pathogens. Altered colonic pH could affect metabolic activity, absorption of short-chain fatty acids (SCFA), and composition of gut microflora and mucosal cell proliferation [34]. Walker *et al.* observed that even a single unit change in pH affected the host microbial community composition as well as production of SCFA, especially among butyrate-producing species [35], a group within which *Fusobacterium* is included [36]–[39]. While most bile acids aid solubilization of lipids and facilitate nutrient absorption, some are transformed by intestinal bacteria into toxic secondary bile acids in the colon [40]. Thus, the transformation of secondary bile acids in the colon could have cytotoxic effects that may contribute to various gastrointestinal diseases including colorectal cancer [41]–[43]. Therefore, it is possible that *Fusobacterium* is a commensal that gains from the disruption of intestinal homeostasis as a result of the presence of adenoma or cancer. We evaluated the relationship between *Fusobacterium* species and adenoma size and frequency. However, there were no significant relationships observed between *Fusobacterium* and adenoma size (small, medium and large) or number of adenomas, suggesting that the abundance *Fusobacterium* in colonic mucosa is not impacted by adenoma size or frequency. These results are consistent with the findings of Castellarin *et al.* [10] who observed no association between *Fusobacterium* abundance and colorectal cancer tumor stage, site, treatment, patient age or survival.

Our goal was to study adenomas, precursors to colorectal cancer, to assess whether an association with *Fusobacterium* was present. We found an increased abundance of *Fusobacterium* among adenoma cases compared to controls. Our findings that *Fusobacterium* is associated with colorectal adenomas implicate its possible involvement early in carcinogenesis. Our work builds on the findings of previous studies reporting an association between *Fusobacterium* and colorectal cancer. [8], [10], [11]. Our observation of a positive correlation between *Fusobacterium* and mucosal inflammation in adenoma cases suggests that this relationship may possibly be mediated by inflammation. However, given the complex environment in which *Fusobacterium* exists with other bacteria [18], we exercise caution in assigning causality as we recognize that more mechanistic studies are needed to elucidate such an association. Recently the alpha-bug and driver-passenger models of CRC proposed by *Sears et al.* and Tjalsma *et al.* respectively [44], [45] defined bacterial drivers (or alpha bugs) as gut bacteria with pro-carcinogenic features such as possession of virulence factors, ability to directly modulate mucosal immune responses and ability to alter bacterial community composition to favor proliferation of opportunistic bacteria (passengers). Thus, under these models *Fusobacterium* could be an important player. Future studies in animal models could help tease apart the precise contribution of *Fusobacterium* and other bacteria to colorectal carcinogenesis.

## Materials and Methods

### Ethics Statement

Institutional approval was provided by University of North Carolina, School of Medicine Institutional Review Board.

### Study Population and Sampling

Two sets of human samples were analyzed in this study. The first group of subjects was drawn from participants in the Diet and Health Study V who underwent routine colonoscopy screening at UNC Hospitals, Chapel Hill, NC. Eligible subjects 30 years of age or older gave written informed consent to provide colorectal biopsies as well as a phone interview involving questions about diet and lifestyle. At the time of the colonoscopy procedure, the research assistant obtained anthropometric measures to determine body mass index (BMI) and waist–hip ratio (WHR) [13], [20]. Biopsy samples from a total of 115 randomly selected subjects (48 adenoma cases and 67 non-adenoma controls) were used in this study. Subjects with known or suspected colorectal cancer or with inadequate colon prep were excluded from the study. Before the endoscopy procedure was performed, biopsies were taken 8–12 cm from the anal verge of the normal rectal mucosa, and immediately flash frozen in liquid nitrogen. Biopsies were stored at −80°C. Participants with reported adenomas were classified as “cases” and those with no adenomas as “controls” [20].

The second group of samples was made up of de-identified matched tumor and normal tissue biopsies from 10 patients with colorectal cancer. Samples were obtained from UNC Tissue Procurement Facility to confirm previously reported studies.

### Fusobacterium Culture

*Fusobacterium nucleatum* subsp. *nucleatum* (ATCC® 25586™) was obtained from the American Type Culture Collection (ATCC) and cultured according to their instructions for use as a positive control. The strain was grown on Reinforced Clostridial Medium (Difco, Becton Dickinson, Franklin Lakes, NJ) under anaerobic conditions at 37°C.

### DNA Extraction

DNA was extracted from normal rectal mucosal biopsies as well as matched tumor/normal tissue using the Qiagen DNeasy Blood and Tissue Kit (Cat# 69504) and a modified protocol with lysozyme and bead-beating [13], [20]. Pure cultures of *F. nucleatum* were centrifuged, re-suspended in kit-provided lysis buffer, and DNA extraction was performed using the same extraction method used for biopsies.

### Pyrosequencing of Colorectal Tumors and Matched Normal Tissue

For CRC tumor and matched normal tissue, the pyrosequencing protocol is as described previously [20]. Briefly, the bacterial 16S rRNA gene was amplified from each DNA sample using barcode-tagged universal 16S primers that span the V1–V3 variable regions. PCR amplification and detection were performed using 454 GS FLX Titanium technology in the UNC Microbiome Core Facility. The primers were composed of the Roche Titanium Fusion Primer A (5′-CGTATCGCCTCCCTCGCGCCATCAG-3′), a 10 bp MID barcode (Roche, Indianapolis, IN) unique to each of the samples and the universal bacteria primer 27F (5′-AGAGTTTGATCCTGGCTCAG-3′)The reverse primers were composed of the Roche Titanium Primer B (5′-CTATGCGCCTTGCCAGCCCGCTCAG-3′) the identical 10 bp MID as the forward primer and the reverse bacteria primer 338R (5′-TGCTGCCTCCCGTAGGAGT-3′) [46].

### Analysis of Bacterial Pyrosequencing Data from CRC Tissue

Analysis of deep sequencing data from CRC tumor and matched normal tissue was performed using QIIME [47]. Briefly, the combined raw sequencing data as well as metadata describing the samples were de-multiplexed and filtered for quality control, following which the data were denoised by PyroNoise and sequences were grouped into OTUs (Operational Taxonomic Units) at 97% sequence similarity to approximate genus-level phylotypes. After taxonomic assignment of OTUs, sequences were aligned for phylogenetic analysis as well as alpha and beta diversity calculations. In addition to t-tests, Wilcoxon-rank sum test was used to compare bacterial abundance and diversity in CRC tumor versus normal tissue.

### Quantitative Real-Time PCR (qPCR)

For mucosal samples from adenoma cases and non-adenoma controls as well as CRC tumors and matching normal tissue, qPCR was performed to quantify the abundance of *Fusobacterium* species. A standard curve was generated by amplifying a16S rDNA region of *F. nucleatum (*ATCC® 25586™*)* using *Fusobacterium*-specific primers [48]. Concentration of the PCR product was measured by absorbance and the number of fragment copies was calculated using the following formula:
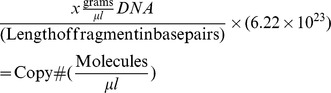



Copy number was adjusted to a starting concentration of 1.00×10^10^ and serial dilutions were performed to create nine standards. 25 µl reactions were prepared containing template DNA, 10µM primer mix, and Fast-SYBR Green Master Mix (Applied Biosystems). The qPCR was performed with an annealing temperature of 60°C for 40 cycles. Finally, the copy number was calculated based on the standard curve, which was adjusted to a starting DNA concentration of 50 ng/µL using the following formula to the unadjusted values:
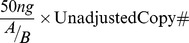
where *A* is the concentration of the template DNA and B is the dilution, 1∶10.

### Reverse Transcriptase PCR (RT-PCR) for Local Inflammatory Cytokines in Adenoma Cases and Non-adenoma Controls

RT-PCR was performed to assess mRNA expression of inflammatory cytokines IL-6, IL-10, IL-12, IL-17 and TNF-α using ready-to-use optimized primers (SA Biosciences). Expression of each inflammatory cytokine was assessed relative to the housekeeping gene hydroxymethylbilane synthase (HMBS). The qPCR was performed using SYBR Green Master Mix (Applied Biosystems) and each sample was run in duplicate. qPCR results were normalized using the expression of the HMBS gene [49].

### Fluorescence *in situ* Hybridization (FISH)

FISH was performed on paraffin sections of mucosal biopsies fixed with Carnoy’s fixative using a universal bacteria probe (EUB388) and a *Fusobacterium*-specific probe. These assays used a previously described protocol [13]. Pure cultures of *E. Coli* and *Fusobacterium* were used as positive controls.

### Statistical Analysis

Mean and standard errors were computed for continuous variables. Comparison of continuous variables such as age (years), waist hip-ratio, body mass index (Kg/m^2^), alcohol (grams/day) calories (Kcal/day), fat (grams/day), red meat (ounces/day) and dietary fiber intake (grams/day) between adenoma cases and non-adenoma controls were made using t-tests. Categorical variables for CRC subjects (tumor location, disease stage, adjuvant therapy) were compared by Fisher’s Exact Test. *Fusobacterium* copy numbers were assessed for normality and log transformed. Mean log *Fusobacterium* abundance was compared between case and control subjects using t-test. The distribution of *Fusobacterium* among control subjects were used to generate tertile values for logistic regression analysis. The lowest tertile of *Fusobacterium* abundance was considered as the reference. Correlation of *Fusobacterium* and local gene expression of TNF-α, IL-17, IL-12, IL-10 and IL-6 were assessed by Spearman's correlation coefficient.

## Supporting Information

Table S1 **Relationship between** ***Fusobacterium*** **abundance and adenoma size.**
(DOCX)
