# Supplementary material for: Fusobacterium Is Associated with Colorectal Adenomas
Source: PLoS One. 2013 Jan 15;8(1):e53653. doi: 10.1371/journal.pone.0053653 (PMC3546075; doi:10.1371/journal.pone.0053653)
Supplement: Table S1 — Relationship between Fusobacterium abundance and adenoma size. (DOCX) [file pone.0053653.s001.docx]

**Table S1: Relationship between *Fusobacterium* abundance and adenoma size.**

| **Adenoma size** | **Fusobacterium Abundance * (mean)** |
| --- | --- |
| **Small (1-5mm)** | **8.37 ± 0.42** |
| **Medium (6-10mm)** | **8.45 ± 1.03** |
| **Large (>10mm)** | **8.86 ± 0.38** |

*The abundance of *Fusobacterium* in mucosal biopsies was determined as described in the methods. Table shows log copy number of *Fusobacterium* among different sizes of adenomas in case subjects.
